# Neural evidence for the use of digit-image mnemonic in a superior memorist: an fMRI study

**DOI:** 10.3389/fnhum.2015.00109

**Published:** 2015-03-05

**Authors:** Li-Jun Yin, Yu-Ting Lou, Ming-Xia Fan, Zhao-Xin Wang, Yi Hu

**Affiliations:** ^1^Key Laboratory of Brain Functional Genomics, Ministry of Education, Shanghai Key Laboratory of Brain Functional Genomics, Institute of Cognitive Neuroscience, School of Psychology and Cognitive Science, East China Normal UniversityShanghai, China; ^2^Shanghai Key Laboratory of MRI, East China Normal UniversityShanghai, China

**Keywords:** superior memory performance, mnemonic, fMRI, episodic memory, working memory

## Abstract

Some superior memorists demonstrated exceptional memory for reciting a large body of information. The underlying neural correlates, however, are seldom addressed. C.L., the current holder of *Guinness World Record* for reciting 67,890 digits in π, participated in this functional magnetic resonance imaging (fMRI) study. Thirteen participants without any mnemonics training were included as controls. Our previous studies suggested that C.L. used a digit-image mnemonic in studying and recalling lists of digits, namely associating 2-digit groups of “00” to “99” with images and generating vivid stories out of them (Hu et al., 2009). Thus, 2-digit condition was included, with 1-digit numbers and letters as control conditions. We hypothesized that 2-digit condition in C.L. should elicit the strongest activity in the brain regions which are associated with his mnemonic. Functional MRI results revealed that bilateral frontal poles (FPs, BA10), left superior parietal lobule (SPL), left premotor cortex (PMC), and left dorsolateral prefrontal cortex (DLPFC), were more engaged in both the study and recall phase of 2-digit condition for C.L. relative to controls. Moreover, the left middle/inferior frontal gyri (M/IFG) and intraparietal sulci (IPS) were less engaged in the study phase of 2-digit condition for C.L. (vs. controls). These results suggested that C.L. relied more on brain regions that are associated with episodic memory other than verbal rehearsal while he used his mnemonic strategies. This study supported theoretical accounts of restructured cognitive mechanisms for the acquisition of superior memory performance.

## Introduction

People are curious about the ability of recalling a large body of information, such as the astonishing performance of reciting tens of thousands of digits after decimal in π. Rajan had the *Guiness World Record* of 31,811 digits in 1981, and Tomoyori broke the record by 40,000 digits in 1987. In 2005, C.L. set the current record of 67,890 digits, and still holds it now. The behavioral mechanisms of the superior performance in these memorists have been extensively explored within laboratory contexts (Thompson et al., 1991, 1993; Ericsson et al., 2004; Takahashi et al., 2006). It was reported that memorists can use various mnemonics, such as chunking, imaging, story-telling and the “Method of Loci.” The earliest study on expertise (i.e., chess players) have revealed that experts can increase their memory performance on domain-specific tasks by chunking the meaningless stimuli together (e.g., chess pieces) (Chase and Simon, 1973), in order to circumvent the limit imposed by the magical number 7 (Miller, 1956). The chunking explanation was later developed into the skilled memory theory (SKE). The theory suggests that the memorists are able to convert randomized items into meaningful codes (e.g., words or images) and to use the retrieval cues in a hierarchical way for subsequent recall (Chase and Ericsson, 1981, 1982). Lately, the theory of long-term working memory (LTWM) (Ericsson and Kintsch, 1995) and template theory (Gobet and Simon, 1996) propose that memorists as well as experts in domains can access information in long-term memory (LTM) when they are performing working memory (WM) tasks, and thus demonstrating better memory performance than the average people who rely mainly on verbal rehearsal.

However, only a few neuroimaging studies have explored the neural basis of memory expertise. In 2003, a group of superior memorists from the World Memory Championship were recruited, and their brain activations during encoding materials with varying degrees of familiarity (i.e., digits, faces, and snowflakes) were investigated (Maguire et al., 2003). The results showed that the use of mnemonics in the encoding process for memorists was associated with activations of the left superior parietal lobule (SPL), retrosplenial and right posterior hippocampus, raising the possibility that specific neural correlates were involved when using mnemonics. However, their findings are not conclusive, given that the memorists in their study used various methods (e.g., “Method of Loci” as well as associating digit stimuli with images). A recent case study on a memorist (PI) showed increased activities in middle frontal gyrus and dorsolateral prefrontal cortex (DLPFC) while he was asked to recall the first 540 digits of π using the “Method of Loci” (Raz et al., 2009). However, the lack of control group in this study led to low validity of the conclusion drawn from the results. Recently, another group of top-50 superior memorists from the World Memory Championship were investigated with tasks of recalling of binary digits which have been learnt either days before or right before retrieval (Boris, 2013), but no significant activation were found in the brain regions that are related to verbal working memory (e.g., frontal brain regions). Other studies investigated the effects of mnemonics training in normal population, and different neural correlates have been reported. For example, the training of “Method of Loci” could induce the activations in the left occipito-parietal cortex, left DLPFC and hippocampus (Nyberg et al., 2003; Valenzuela et al., 2003; Kondo et al., 2005). The mathematic coding strategy was associated with activations in lateral prefrontal cortex (Bor and Owen, 2007). The visual working memory strategy evoked the activities in the posterior parietal, bilateral/dorsolateral prefrontal and occipito-temporal cortices (Moore et al., 2006). The rote learning strategy was associated with activations in bilateral DLPFC (Maestu et al., 2003). The verbal strategy was associated with activities in the right medial temporal lobe (Sanfratello et al., 2014). Based on LTWM and template theory, a more recent review of neuroimaging studies described a two-physiological-stage framework for expertise acquisition: (1) decreased activations in the WM-related brain areas (e.g., the prefrontal and parietal areas) were corresponded to the chunking process; (2) increased activations in the LTM-related brain areas were corresponded to the development of knowledge structure as retrieval structure or templates (Guida et al., 2012). Clearly, these findings suggested that different mnemonics involved different neural correlates.

In the current fMRI study, we investigated the neural correlates of the mnemonic used by C.L., who is the current holder of Guinness World Record for reciting digits of π. In our previous behavioral studies (Hu et al., 2009; Hu and Ericsson, 2012), C.L. reported that he used a digit-image mnemonic to memorize digit sequences, which included automatically converting each of the two-digit groups (from “00” to “99”) into an image. For example, “72,” “44,” and “79” were converted into “penguin,” “sailing boat,” and “balloon,” respectively. After that, he generated vivid stories out of the images, such as “a penguin is sitting in the sailing boat, with balloons in her hand.” The mnemonic, together with other memory techniques (e.g., the “Method of Loci”), were used when memorizing lists of digits. Thus, 2-digit numbers were used as the target condition in this study. Participants without any mnemonic trainings were included in the present study as controls. When compared to C.L., the 2-digit condition should be more difficult for the control group. To address this possible confounding factor, we presented each 2-digit stimulus for 2 s, in which C.L.'s memory performance were found to be similar to that of normal people in our previous study (Hu et al., 2009). According to the two-physiological-stages framework (Guida et al., 2012), the mnemonics used by C.L. while memorizing 2-digit condition might be associated with the episodic memory. But it is also possible that when using the mnemonic, he may use verbal strategy rather than episodic-like memories to express the stories. To rule out this possibility, the letter and 1-digit conditions were also included as control conditions. It is unlikely that C.L. can use the same mnemonic to memorize letters and 1-digit numbers without extensive practice. Therefore, we hypothesized that in comparison to the control participants, C.L.'s neural regions associated with episodic-like memories would be more engaged during 2-digit number encoding relative to control conditions.

## Material and methods

### Participants

The superior memorist, C.L. (aged: 28), and 13 male graduate students from the East China Normal University participated in this experiment as controls. No known mnemonic trainings were reported by the controls. Data from two controls were excluded due to excessive head movement during fMRI scanning, and the average age of the remaining 11 controls was 24 years (age range: 21–26 years; *SD* = 1.6 years). All were right-handed, with normal or corrected-to-normal vision. No known psychological, neurological disorder, history of head trauma was reported. Participants were paid in compensation for their effort and time. Written consents were obtained from all participants. The Ethics Committee of the East China Normal University approved this study.

### Experimental procedure

**Figure 1:**
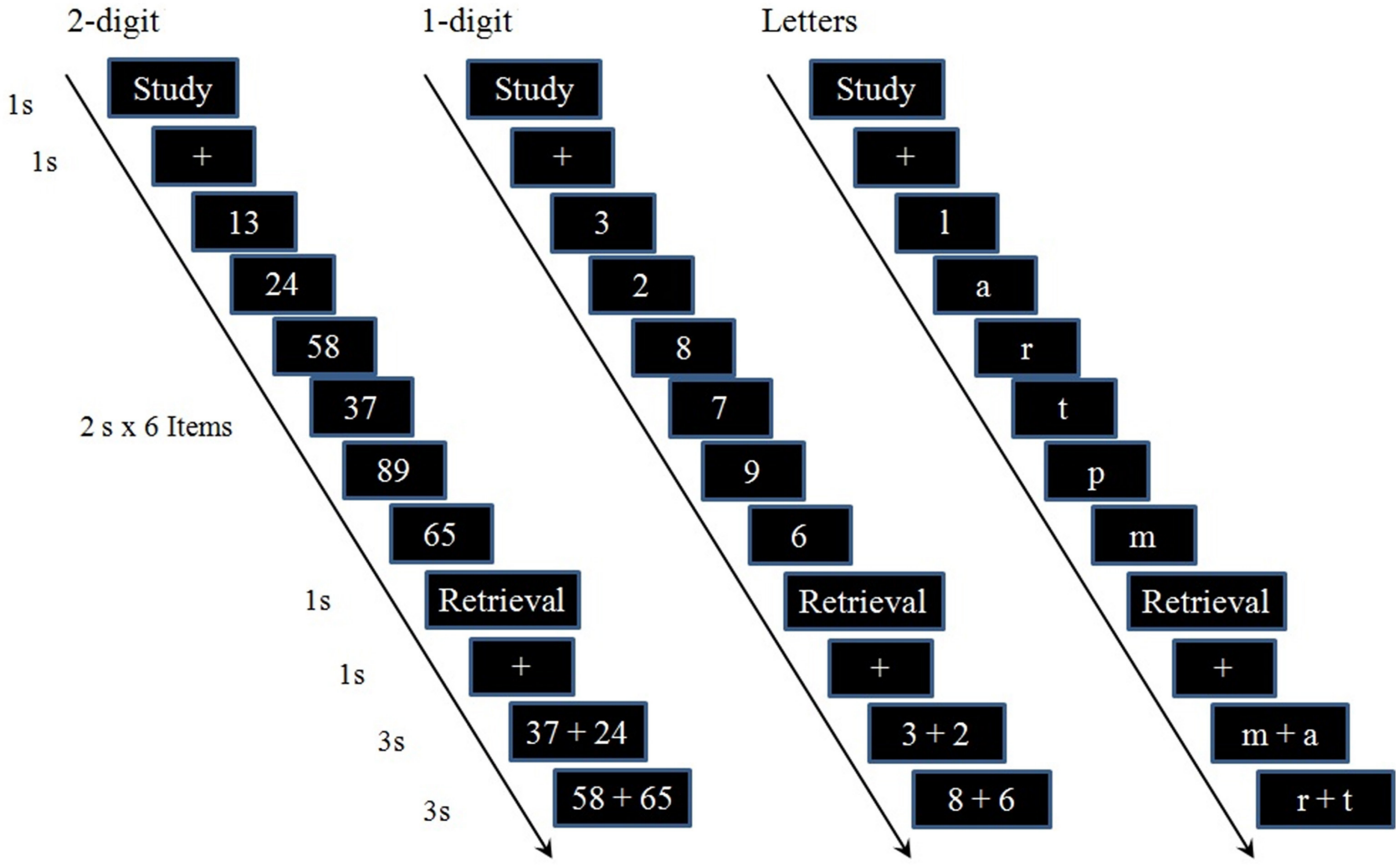
**Example trials of the three conditions (2-digit, 1-digit, and letter) used in the fMRI session**. In the encoding phase, participants were asked to memorize a sequence of six items which were presented sequentially at the rate of 2 s per item. In the recall phase, two items were presented at both sides of the screen, and participants were asked to judge which one came earlier by pressing the left or the right button. There were three judgments in one trial. Each trial lasted 22 s. The inter-trial interval (ITI) was set at 10 s. All cues are in Chinese.

An event-related fMRI design was adopted. There were three functional runs with 30 trials each. The learning stimuli used in each run included one out of three conditions, i.e., two-digit numbers, one-digit number, or letter (Figure 1). All stimuli were presented in white with a black background and were presented with an fMRI compatible goggles system (Invivo Co., USA).

In a trial (Figure 1), a cue of “Study” (1 s, in Chinese) was first presented, and followed by a fixation of “+” (1 s). Then, a sequence of six items was presented sequentially at the rate of 2 s per item. Participants were asked to memorize these items in their original order. Then, a cue of “retrieval” (1 s, in Chinese) was presented. After a fixation of “+” (1 s), two of these six items would be presented for 2 s, one at the left side of the fixation and one at the right. Participants were asked to judge which one came earlier by pressing the left or the right button using a 2-button fMRI compatible response box. Another two items were then presented for 2 s for the second judgment. Each trial lasted 22 s. The inter-trial interval (ITI) was set at 10 s.

After the fMRI session, participants were asked to describe how they performed these tasks and whether they adopted special memory strategies to aid in their encoding. No special method but verbal rehearsal was reported by controls, while C.L. reported that he used the digit-image mnemonic in the 2-digit condition only.

### Data collection and analyses

The scanning was conducted in a 3-Tesla Siemens Trio MRI scanner, including three 964-s long functional runs (30 22 s-long trials with 10-s ITI, and 4-s dummy images) and 1 anatomical run. For functional images, 35 axial slices (Field of view = 240 × 240 mm^2^, matrix = 64 × 64, in-plane resolution = 3.75 × 3.75 mm^2^, thickness = 4 mm, without gap) covering the whole brain were obtained using a T2^*^-weighted echo planar imaging (EPI) sequence (*TR* = 2000 ms, *TE* = 30 ms, flip angle = 90°). A high-resolution structural image for each participant was also acquired using 3D MRI sequences for anatomical co-registration and normalization (*TR* = 1900 ms, *TE* = 3.43 ms, flip angle = 7°, matrix = 256 × 256, FOV = 240 × 240 mm^2^, slice thickness = 1 mm).

SPM8 was adopted for data processing and analyses with standard procedure (Wellcome Department of Cognitive Neurology, London, UK; http://www.fil.ion.ucl.ac.uk/spm/). Behavioral and imaging data from two control participants was excluded due to excessive head motion. (Friston et al., 1994). One-sample *t*-test was then used to identify regions differentially engaged by C.L. and controls in different conditions. Comparisons between the contrasts of 2-digit and letter conditions of C.L. and controls (interaction) were also performed by *t*-tests. All comparisons between different conditions and groups were masked by the combined activation maps of relevant conditions vs. fixation. Brain regions with voxel-wise threshold of *p* < 0.001 and at least 100 consecutive voxels were reported for exploratory purpose, while only brain regions that survive family-wise error (FWE) correction at cluster level (*p* < 0.05) were discussed.

For the behavioral data, the number of correct responses was calculated as the memory performance. To compare C.L.'s performance with that of the control group, we used a modified *t*-test for one sample (Crawford and Howell, 1998).

## Results

### Behavioral results

There was no significant difference between the number of correct responses of C.L. (*N*_2-digit_ = 44; *N*_1-digit_ = 56; *N*_letter_ = 56) and that of control participants (*N*_2-digit_ = 44.9, *SD* = 5.2; *N*_1-digit_ = 53.9, *SD* = 3.3; *N*_letter_ = 50, *SD* = 4.6) for all three conditions (*p*s > 0.24). In the controls, a main effect of condition was found in the repeated measures ANOVA [F_(2, 20)_ = 20, *p* < 0.001, η ^2^ = 0.667]. *Post-hoc* analyses revealed that there were significant differences between the three conditions (*ps* < 0.05, Bonferroni correction).

### Imaging results: study phase

#### Regions engaged by C.L.

**Figure 2:**
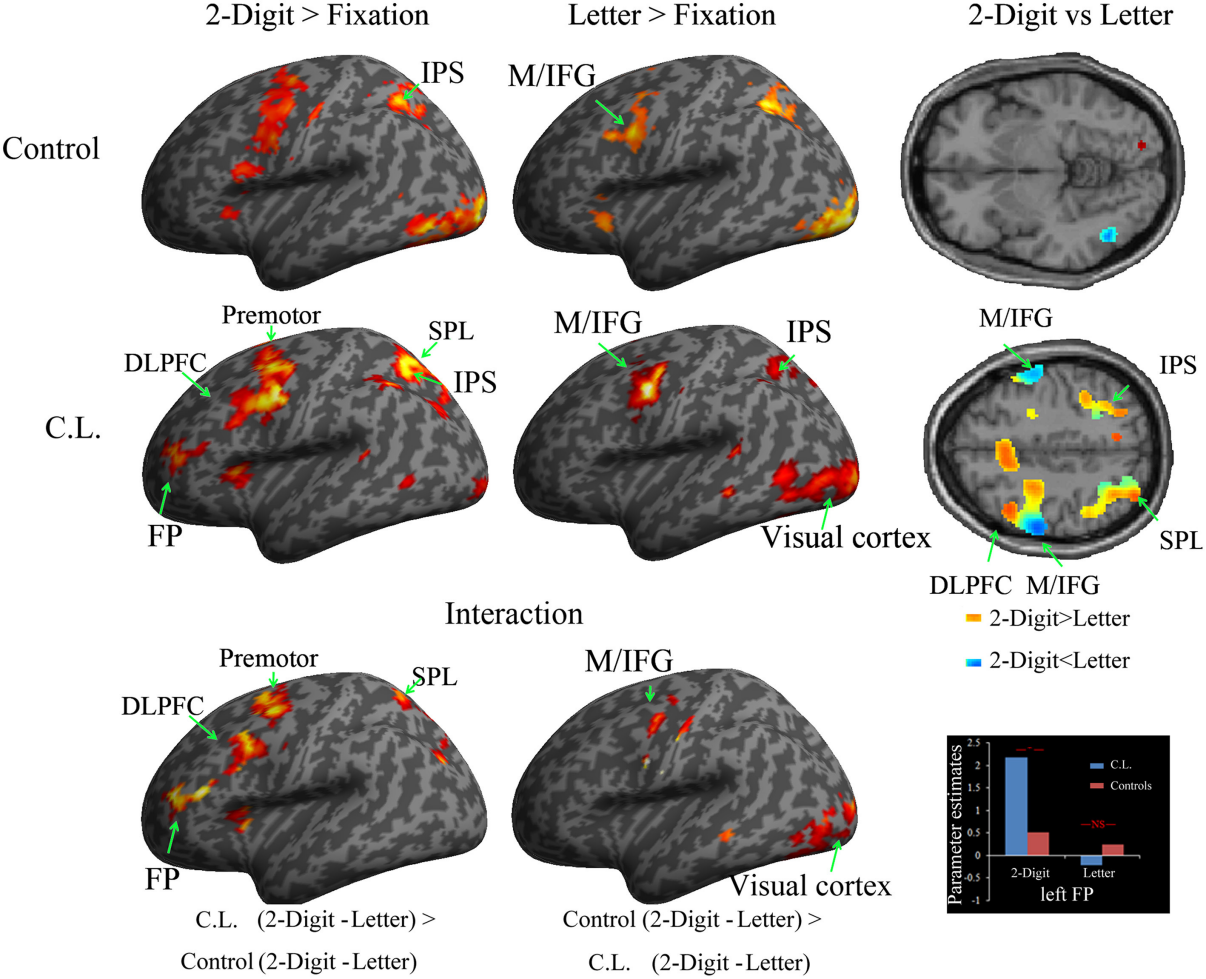
**Brain regions (left hemisphere) recruited in the study phase (*p* < 0.001, uncorrected, *k* >100) were displayed on a standard inflated brain**. The direct comparisons between 2-digit and letter conditions were displayed on the right panel (axial plane). The FP, premotor cortex, SPL, and DLPFC were more engaged in the 2-digit condition than in the letter condition of C.L., while the M/IFG were more engaged in the letter condition than in the 2-digit condition. The interactions were displayed at the bottom panel. The parameter estimates of the left FP were also displayed. Abbreviations: FP, frontal pole; DLPFC, dorsolateral prefrontal cortex; SPL, superior parietal lobule; IPS, intraparietal sulcus; M/IFG, middle/inferior frontal gyri.

By calculating the contrast for each condition vs. fixation, the analyses revealed that a number of brain regions were commonly recruited in the 2-digit, letter, and 1-digit conditions vs. fixation (Tables S1–S3). These included the bilateral middle/inferior frontal gyri (M/IFG; MNI coordinates: −52 2 48; 60 0 44), premotor cortex (PMC, −26 0 50; 38 6 60), insula (−32 18 10, 38 16 8), parietal cortices (−36 -62 58, 40 -42 44), supplementary motor area/anterior cingulate cortex (SMA/ACC, −2 8 56), and visual cortices. However, the bilateral frontal poles (FP, BA10, −38 46 14; 30 42 6) were found to be activated only in the 2-digit condition, but not in the 1-digit and letter conditions (Figure 2, Supplementary Figure S1).

Direct comparison between the 2-digit condition and the letter condition showed that several brain regions showed greater neural recruitment in the 2-digit condition, including bilateral FPs (−40 50 16, 42 48 18), PMC (−26 2 54, 38 6 58), left SPL (−10 −66 58), left insula (−34 16 8), and ACC/SMA (−6 18 42). The opposite comparison revealed that bilateral M/IFG (−56 −6 44, 60 −4 40) and visual cortices (−24 −96 10, 38 −92 −4) were more engaged in the letter condition (Figure 2).

Direct comparison between the 2-digit condition and the 1-digit condition showed that several brain regions were more engaged in the 2-digit condition, including bilateral FPs (−40 50 12, 32 44 8), PMC (−26 2 56, 30 14 62), insula (−34 16 8; 38 16 4), left SPL (−10 −68 58), and ACC/SMA (2 20 42). On the other hand, left visual cortices (−22 −102 10) were more engaged by the 1-digit condition. Note that bilateral M/IFG (−58 −12 48, 89 voxels, 60 −10 42, 25 voxels) were more engaged in the 1-digit condition with a lenient threshold (*p* < 0.001, *k* > 20).

#### Regions engaged by control participants

A number of brain regions were activated in the 2-digit, letter, and 1-digit conditions vs. fixation (Tables S1–S3, Figure 2), including the bilateral M/IFG (−42 0 34; 46 6 28), PMC (−32 0 66; 28 4 56), insula (−30 20 −2, 34 20 2), IPS (−30 −52 44, 34 −46 48), and visual cortices (−38 −80 −6; 26 −88 −2).

The direct comparison between the 2-digit and letter conditions revealed greater neural recruitment only in the right visual cortices (22 −86 4) in the 2-digit condition, while the left M/IFG (−43 −15 52) and left visual cortices (−46 −64 −8) were more engaged in the letter condition (Figure 2). Regarding the comparison between 2-digit condition and 1-digit condition, the bilateral visual cortices (−24 −90 2; 24 −84 2), SMA/ACC (−8 22 44), the right PMC (26 14 50), and right IPS (36 −42 34) were more engaged in the 2-digit condition, while no brain region was more engaged in the 1-digit condition.

#### Interaction (comparisons of the 2-digit vs. letter conditions)

Further comparisons of the contrasts of 2-digit condition vs. the letter condition between C.L. and Controls were performed (Table 1). The results revealed significant interactions in the bilateral FPs (−40 46 14; 30 42 4), PMC (−26 0 50; 32 18 60), left SPL (−32 −84 36) and left insula (−32 10 6), where C.L. displayed stronger activations in the 2-digit condition than in the letter condition (Figure 2, bottom panel, left). Significant interactions were also found in the bilateral M/IFG (−56 −10 44; 64 −6 38) and left visual cortex (−20 −94 8), where C.L. displayed weaker activations in the letter condition than in the 2-digit condition (Figure 2, bottom panel, middle). Direct comparisons of the 2-digit and letter conditions between C.L. and controls were also performed, and similar results were found (Figure 2).

**Table 1:** **Direct comparison of the study phase of 2-digit condition vs. letter condition between C.L. and controls**.

**Hem**	**Maxima location**	**MNI Coordinates**			***T*_max_**
		**x**	**y**	**z**	
**C.L. (2-DIGIT – LETTER) > CONTROLS (2-DIGIT – LETTER)**					
L	Frontal Pole	−40	46	14	17.7^*^
	Premotor cortex	−26	0	50	15.4^*^
	DLPFC	−38	14	44	12.6^*^
	Insula	−32	10	6	12.9^*^
	Posterior parietal lobe	−28	−66	62	11.0
	Superior parietal lobule	−32	−84	36	12.6^*^
C	ACC/SMA	−8	16	42	7.7
R	Frontal Pole	30	42	4	20.2^*^
	Premotor cortex	32	18	60	7.9
**CONTROLS (2-DIGIT – LETTER) > C.L. (2-DIGIT – LETTER)**					
L	Middle frontal gyrus	−56	−10	44	16.7^*^
	Visual cortex	−20	−94	88	14.7^*^
R	Middle frontal gyrus	64	−6	38	13.8
	Intraparietal sulcus	26	−44	52	6.2

*The Voxel-wised threshold was set p < 0.001, uncorrected, spatial extent > 100 voxels, masked by the combined activation maps during studying phase of the 2-number condition of C.L. and of controls*.

* *FWE corrected at cluster level (p < 0.05). Abbreviation: DLPFC, dorsolateral prefrontal cortex; ACC/SMA, anterior cingulate cortex/supplementary motor area*.

### Imaging results: recall phase

#### Regions engaged by C.L.

**Figure 3:**
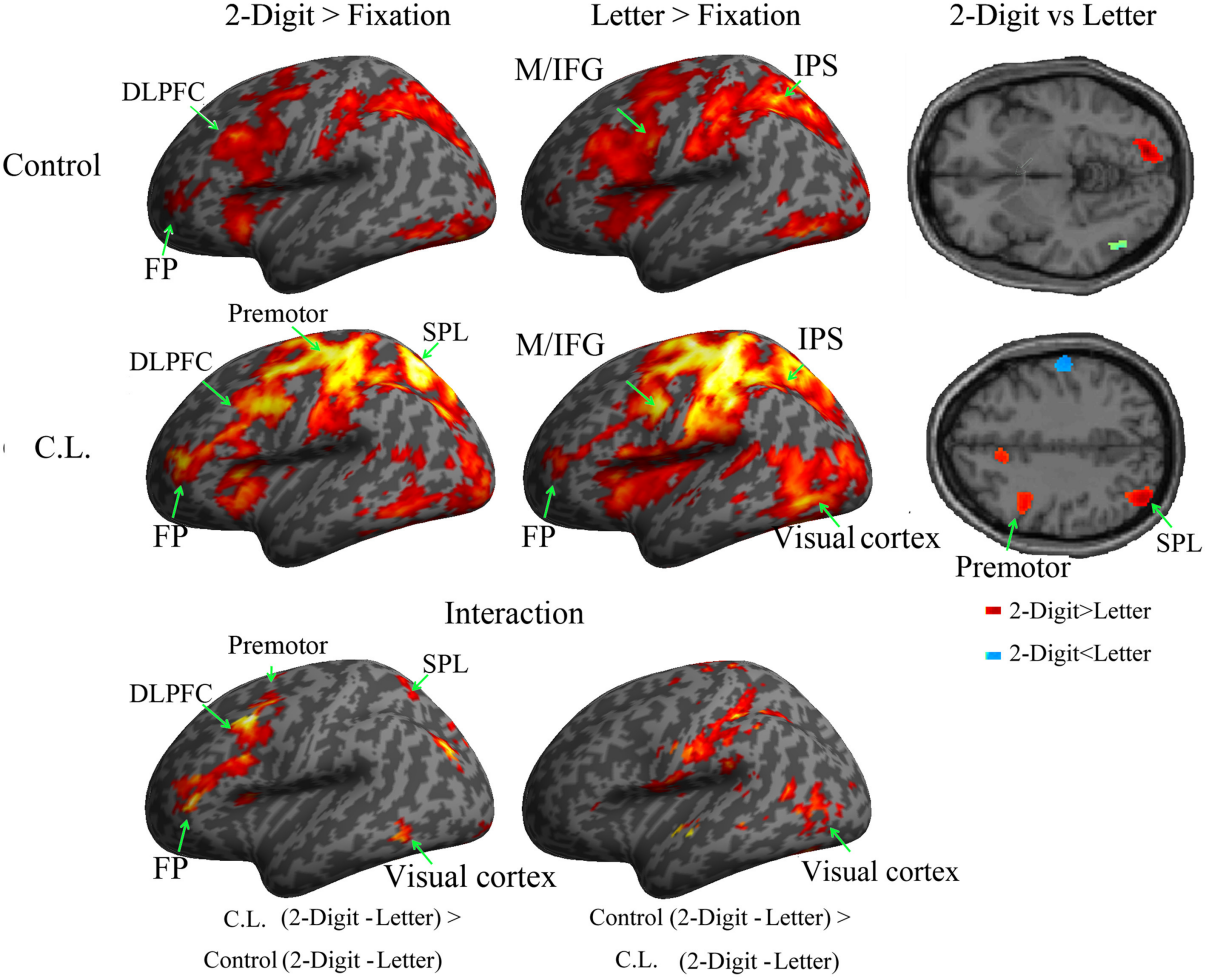
**Brain regions (left hemisphere) recruited in the recall phase (*p* < 0.001, uncorrected, *k* >100) were displayed on a standard inflated brain**. The direct comparisons between 2-digit and letter conditions of C.L. as well as the controls were displayed on the right panel (axial plane). The FP, DLPFC, premotor cortex, and SPL were more engaged in the 2-digit condition than in the letter condition of C.L. The interactions were displayed at the bottom panel. Abbreviations: FP, frontal pole; DLPFC, dorsolateral prefrontal cortex; SPL, superior parietal lobule; IPS, intraparietal sulcus; M/IFG, middle/inferior frontal gyri.

A number of brain regions were commonly activated in the 2-digit, letter, as well as 1-digit conditions, including bilateral FPs (−36 44 10, 30 44 4), M/IFG (−52 8 38; 60 14 20), PMC (−38 −6 62; 38 2 58), insula (-30 22 −2, 38 20 −4), IPS (−30 −64 52, 38 −60 40), primary sensory/motor cortex (−46 −34 56, 46 −40 56), basal ganglia (−28 −10 2, 26 −2 6), visual cortices (−36 −78 10, 38 −86 −8), SMA/ACC (4 22 42) and the left SPL (−14 −76 54) (Tables S4–S6, Figure 3).

Direct comparison between 2-digit and letter conditions showed that several brain regions were more engaged in the 2-digit condition (Figure 3), including the left PMC (−38 14 46), left SPL (−36 −78 40), SMA/ACC (−6 18 44), and bilateral visual cortices (−16 −98 −4; 20 −96 −4). Note that bilateral FPs (−36 44 10; 30 52 −4) were also activated with a lenient threshold (*p* < 0.005). No brain region was more engaged in the letter condition.

The results of direct comparison between 2-digit and 1-digit conditions showed that several brain regions were more engaged in the 2-digit condition, including bilateral FPs (−40 50 10; 32 48 −2), the left PMC (−32 6 58), left SPL (−32 −66 54), SMA/ACC (4 24 40), and bilateral visual cortices (−22 −94 −2; 16 −98 −8). No brain region was more engaged in the 1-digit condition.

#### Regions engaged by control participants

A number of brain regions were commonly recruited in the 2-digit, letter, and 1-digit conditions, including the bilateral FPs (−38 48 4, 30 56 0), M/IFG (−52 14 36; 46 10 32), PMC (−30 −4 60; 28 14 58), insula (−32 18 −2, 32 28 −2), IPS (−36 −52 46, 24 −60 48), primary sensory/motor cortex (−48 −38 50, 44 −36 42), basal ganglia (−22 −16 0, 18 −8 0), visual cortices (−24 −92 −8, 24 −86 −12), and the SMA/ACC (−2 22 48) (Tables S4–S6, Figure 3).

Direct comparison between 2-digit and letter conditions showed that several brain regions were more engaged in the 2-digit condition (Figure 3), including the right PMC (30 14 56), SMA/ACC (0 28 44), and right visual cortices (26 −84 −8), while the left primary sensory/motor cortex and the left middle occipital gyrus (−52 −66 −10) were more engaged in the letter condition.

Direct comparison between 2-digit and 1-digit conditions showed that several brain regions were more engaged in the 2-digit condition (Figure 3), including the bilateral FPs (−26 52 2; 28 62 8), PMC (−34 8 46; 34 10 60), SMA/ACC (2 28 44), IPS (−42 −58 46; 36 −72 46), insula (−40 18 −6; 30 28 −2), and visual cortices (−22 −90 −12; 28 −84 −8), while no activation was found in the opposite comparison.

#### Interaction (comparisons between the contrasts of 2-digit vs. letter conditions)

The interaction analysis revealed significant interactions in the bilateral FPs (−36 38 4; 30 44 0), left PMC (−30 4 58), left SPL (−36 −82 38), left insula (−38 14 10), and ACC/SMA (−6 26 36), where C.L. displayed stronger activations in the 2-digit condition than in the letter condition. Significant interaction was also found in the bilateral IPS (−40 −48 45; 40 −50 36), primary sensory/motor cortices (−54 −18 30; 46 −34 34) and visual cortices (Extrastriate cortex, −32 −40 −8; 40 −66 −10), where C.L. displayed stronger activations in the letter condition than in the 2-digit condition. Direct comparisons between C.L. and controls in the recall phase also showed similar results (Table S7).

## Discussion

In the present study, we investigated the neural correlates of brain activations associated with the digit-image mnemonic. The increases and decreases in brain activations observed in C.L., relative to controls, were first discussed. Then these findings were discussed within the contexts of current theories for the superior memory performance.

### The enhanced brain activations in C.L.

#### FPs

The most intriguing finding of the present study is that bilateral FPs were activated only in C.L.'s 2-digit condition in the study phase, but not in the other conditions in both C.L. and controls. Previous studies showed that the FP was neither involved in the encoding processes of both verbal and spatial working memory tasks, such as digit span (Zhang et al., 2004) and the syllabic task (Ischebeck et al., 2008), nor in the encoding of animals (West et al., 2001) or faces (Clark et al., 1998) [animals and faces were used by C.L. in his mnemonics (Hu et al., 2009)]. Thus, the activation in the FPs might not be responsible for the encoding of verbal information. Moreover, in the recall phase across all conditions, activations in the FPs were found in both C.L. and in controls. It has been reported that the FP was involved in retrieving short-term memory (Rugg et al., 1996), long term memory (Buckner et al., 1996), and episodic memory (Tulving et al., 1994), despite the fact that some studies failed to find significant FP activations during retrieval, for example, of words phonologically (Rosen et al., 2000; Perani et al., 2003). These results suggested that FPs are involved in the retrieval processes during recall phase in both C.L. and controls. We assumed that the significantly higher neural activation in the C.L.'s FPs during the study phase might be associated with retrieval of established images linked to 2-digit numbers.

#### SPL and PMC

After controlling for verbal rehearsal, we found that the left SPL of C.L. was activated only in the 2-digit condition, suggesting that the SPL was not responsible for verbal rehearsal. To speculate the role of SPL, we noted that the posterior parietal cortex plays a crucial role in multiple spatial functions, such as spatial awareness (Marshall et al., 2002; Thiebaut De Schotten, 2007), spatial updating (Merriam et al., 2003), short-term and long-term memory representation of spatial information (Saito and Watanabe, 2006; Kesner, 2009), as well as episodic memory retrieval (Hutchinson et al., 2009, 2014). Studies also reported that disruption in parietal function or parietal lesions induced changes in spatial ability, such as decrement of spatial sustained attention (Lee et al., 2013), destabilization of spatial updating (Morris et al., 2007), reflexive spatial attention (Chambers et al., 2007), and spatial neglect (Cochin et al., 1996). Moreover, previous studies revealed that the bilateral parietal cortices played an important role in the evaluation of spatial relativity. For example, activations of the parietal cortices were found in the computation of the correct trajectory or route to be followed while navigating (Calton and Taube, 2009), mental space travel (Ciaramelli et al., 2010) and motor imagery (Fleming et al., 2010). We thus speculate that the activations in the SPL might contribute to the episodic retrieval, spatial association of retrieved images, as well as imagery of vivid stories of the mnemonics used by C.L. Consistently, Maguire also reported activation in the left SPL during the study phase of memorists, most of who used the “Method of Loci” (Maguire et al., 2003).

Another important aspect of C.L.'s mnemonics is to generate vivid stories out of these images by using verbs. The ability of mental imagery is crucial in this process. Activation in the SPL was found in other tasks that require mental imagery. For example, higher activation in the bilateral SPL has been found during serial abacus mental calculation (Tanaka et al., 2002; Chen et al., 2006). Apart from the SPL, the PMC was also found to be involved in pantomiming action of tool (Choi et al., 2001). Previous studies showed that there were dynamic premotor-to-parietal interactions during spatial imagery (Lamm et al., 2001; Li et al., 2005; Szameitat et al., 2007; Sack et al., 2008; Lorey et al., 2011). Therefore, both SPL and PMC might be recruited during C.L.'s spatial imagery.

#### DLPFC

The left DLPFC was also involved during the study phase in the 2-digit condition of C.L., but not in the 1-digit and letter conditions. The DLPFC is believed to play a central role in information manipulation (Smith and Jonides, 1998; Smith et al., 1998, 2001; Zhang et al., 2004; Owen et al., 2005; Zhu et al., 2006). In line with their suggestion, we speculate that the DLPFC in C.L. was to manipulate information and to further generate vivid stories.

#### Recall phase

Results from the recall phase were in line with the findings in the study phase; C.L. displayed enhanced brain activations in bilateral FPs, SPL, and left PMC. Thus, the neural correlates of retrieval were reshaped by his mnemonics, and these episodic images as well as vivid stories were helpful to retrieve those information.

### The decreased brain activations in C.L.

#### M/IFG and IPS

We found that the M/IFG and/or IPS were engaged less in the 2-digit condition of C.L., relative to the 1-digit and letter conditions of C.L., as well as relative to the 2-digit condition of controls. The M/IFG and IPS played crucial roles in the phonological loop or rehearsal verbal information in normal population (Smith and Jonides, 1998; Smith et al., 1998; Owen et al., 2005). We speculate that the relatively less engagements of the M/IFG and IPS of C.L. may imply that C.L. relied less on the rehearsal ability to memorize these 2-digit numbers.

#### Visual cortices

Interestingly, we found that activities in the bilateral visual cortices were weaker in the 2-digit conditions in C.L. than the 1-digit/letter conditions and all conditions of controls. This finding is inconsistent with previous findings (Maguire et al., 2003). In their studies, direct comparisons between the memorists and controls revealed significant activations in the left fusiform cortex, which could be a possible indicator of visual object representations that also found in participants with visual expertise training (Moore et al., 2006) as well as the “Method of Loci” training (Kondo et al., 2005). One explanation is that C.L. spent most of his time to generate the vivid stories, evidenced by his reaction time during study phase (Hu et al., 2009). Thus, he may be disengaged from the visual sensory once the images/words were generated from 2-digit numbers. Although the visual cortices should be also involved, the SPL may play a more important role in his mental imagery. Differed from C.L., controls may still focus on these stimuli to facilitate rehearsal processes. As activations in the visual cortices can be modulated by top-down control (Gilbert and Li, 2013), the disengagement should result in lower activation of the visual cortices. The second possibility is that other than the “Method of Loci,” the meaningful sentences of these stories, but not images, play a more important role in C.L.'s mnemonics. However, he told us in one of interviews that the codes for digits in his memory were vivid images rather than the words. Moreover, brain regions associated with episodic processing were more involved in C.L.'s 2-digit condition. Thus, it seems that this possibility is less likely and needs to be verified in future studies.

#### Recall phase

Results from the recall phase were in line with the findings of the study phase, controls displayed enhanced activations in the bilateral IPS as well as M/IFG than that of 2-digit condition of C.L. (Table S7). These findings supported the idea that the C.L. relied less on his ability of rehearsal when recalling information.

### Neural correlates for the use of digit-image mnemonic

In this fMRI study, we found that C.L. displayed two main patterns in his encoding and retrieval in the 2-digit condition, relative to the letter conditions of C.L. as well as all conditions of controls. First, brain activations that were reported to be related with episodic retrieval, mental imagery, and information manipulation, such as the FPs, SPL, PMC, and DLPFC, were more involved in C.L. These results are in line with C.L.'s self-report. Second, brain regions that were associated with verbal rehearsal, such as the M/IFG and IPS regions, were less involved. These results suggested that verbal rehearsal, as well as verbally expressed stories, might play a secondary role in the use of C.L.'s mnemonic, this possibility cannot be ruled out. Altogether, our studies supported a two-stage framework raised by Guida et al. (2012, 2013). That is, acquisition of expertise would lead to a reduction in activation in the neural regions pertaining to working memory, and a reorganization of brain functions.

Our results can also be explained in the context of the LTWM model. In the LTWM model (Ericsson and Kintsch, 1995), Ericsson described that the processes involved in comprehension, such as episodic text comprehension, result in the construction of retrieval structures and thereby generate the LTWM. In other expertise domains, it has been found that the ability to perform complex calculations in expertise were initially supported by extensive attentional and strategic resources and then gradually replaced by access to the LTWM for familiar materials (Minati and Sigala, 2013). In C.L.'s case, he can use his established episodic memory (i.e., long term memory) as well as mental manipulation (i.e., comprehension by generating vivid stories out of meaningless long digit sequences) to facilitate the encoding and retrieval of 2-digit numbers. The development of the LTWM also existed in other expertise domains, yet different neural correlates may be involved. For example, posterior regions were relatively more involved in various reasoning and problem solving tasks in autistic individuals with savant calendar calculating (Dubischar-Krivec et al., 2014).

Note that we did not find significant activation in the medial temporal lobe (MTL) during the study phase of C.L., even with a very lenient threshold (*p* < 0.1), which has been found activated in Maguire et al.'s (2003) study. One possible explanation is that the MTL plays an important role in the “Method of Loci” as most of their memorists used this method, while the “Method of Loci” could not function in C.L. because C.L. cannot associate 2-digit condition with locations in a fast presentation (i.e., 2 s per pair). We also note that activation in the MTL was not found in Raz et al.'s study in a memorist (Raz et al., 2009), as well as Kondo et al.'s study in normal participants who have been trained to use the “Method of Loci” (Kondo et al., 2005). Further investigation of the role of the MTL in mnemonics, such as the “Method of Loci,” is needed.

C.L. is a native Chinese speaker. Whether the same neural correlates are involved in memorists using the phonetic system digit-image mnemonic deservers more investigation. Note that many of C.L.'s digit-image association are involved with phonetic similarity. For example, 72 is pronounced as “qi er”; and penguin is “qi e” in Chinese, and 79 is “qi jiu,” and a balloon is “qi qiu”; “99” is “jiu jiu,” which is pronounced similar to uncle in Chinese. These similarities may facilitate his association. It seems that the digit-image association used in a phonetic system is more complicated, such as the Dominic System or Major System, yet the story-making mnemonic is similar to the Person-Action-Object System. Thus, we speculate that both common and specific neural correlates are expected in Western memorists using similar mnemonics.

In this case study, the differences in brain activation patterns between C.L. and the controls might be accounted for the anatomical differences between C.L. and controls, despite of the normalization of brain structures. To rule out this possibility, the anatomy image of C.L. was carefully checked by one of the authors (M. F.), a senior radiologist, and no structural abnormality was detected. Moreover, the neural network related to verbal memory, specifically M/IFG, IPS, and ACC/SMA, etc, was almost overlapping between controls and C.L. in the letter/1-digit conditions. These results suggested that this possibility was unlikely.

Another concern is that there are differences in the level of difficulties across the three conditions, resulting in differences in the attentional load. To address this question, the presentation rate was set at 2 s per item, i.e., 1 digit per 2 s for the 2-digit condition, which is difficult for C.L. to encode in the study phase. Our result is consistent with the finding in our previous study where he showed the normal digit memory span at this rate (Hu et al., 2009). The behavioral result shows that there was no significant difference in all three conditions, suggesting that this manipulation was valid and the levels of difficulty were controlled. Moreover, the fact that brain regions, such as the bilateral FPs and left SPL, were exclusively activated in the study phase of the 2-digit condition of C.L., indicating that these differences were not due to differences in attentional load but mnemonics.

## Conclusions

In this study, we found that the episodic-memory-related brain regions, such as FP, SPL and PMC, were more involved in encoding 2-digit numbers for C.L., whereas verbal-related brain regions, such as M/IFG and IPS, were more involved in the controls. It conveyed the information about the function of FP, SPL, and PMC in the short-term memory performance when the digit-image associations were used. The findings supported the re-structured cognitive mechanisms for C.L. who has practiced the mnemonic for a very long period. Future studies may explore the factors constraining the re-arrangement of neural correlates for expertise development, such as the processing speed, the attentional abilities, and the intelligence.

### Conflict of interest statement

The authors declare that the research was conducted in the absence of any commercial or financial relationships that could be construed as a potential conflict of interest.

## Supplementary material

Supplementary Figure S1 **BA 10 is involved in the encoding phase of C.L. left: non-masked activation map. right: activation map masked by BA 10**.
