# Supplementary figures and images for: Neural evidence for the use of digit-image mnemonic in a superior memorist: an fMRI study
Source: Front Hum Neurosci. 2015 Mar 5;9:109. doi: 10.3389/fnhum.2015.00109 (PMC4350403; doi:10.3389/fnhum.2015.00109)

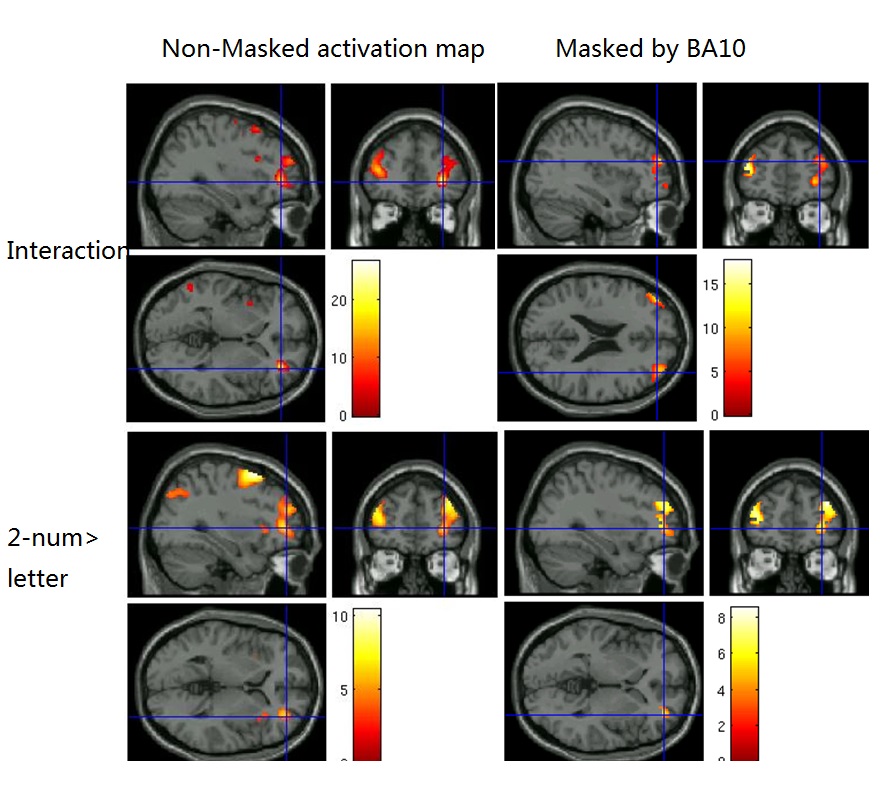

Supplement: Supplementary Figure S1 — BA 10 is involved in the encoding phase of C.L. left: non-masked activation map. right: activation map masked by BA 10. [file Image1.JPEG]
