# Supplementary material for: Neural evidence for the use of digit-image mnemonic in a superior memorist: an fMRI study
Source: Front Hum Neurosci. 2015 Mar 5;9:109. doi: 10.3389/fnhum.2015.00109 (PMC4350403; doi:10.3389/fnhum.2015.00109)
Supplement: Supplementary file 2 [file DataSheet1.DOCX]

| **Supplementary materials**  **Table S1. Regions recruited during the study phase of 2-digit condition** | | | | | | |
| --- | --- | --- | --- | --- | --- | --- |
| **Hem** |  | **Maxima location** | **MNI Coordinates** | | | **T** |
| ***C.L.*** | | | | | | |
| L |  | Frontal pole | -38 | 46 | 14 | 8.8* |
|  |  | Premotor cortex | -26 | 0 | 50 | 11.4* |
|  |  | Middle/inferior frontal gyrus | -52 | 2 | 48 | 13.8* |
|  |  | Insula | -32 | 18 | 10 | 8.5* |
|  |  | Intraparietal sulcus | -36 | -62 | 58 | 11.8* |
|  |  | Superior parietal lobule | -14 | -72 | 58 | 11.4* |
|  |  | Superior temporal gyrus | -68 | -42 | 10 | 7.9 |
|  |  | Visual cortex | -26 | -98 | -2 | 6.2* |
| C |  | ACC/SMA | -2 | 8 | 56 | 10.1* |
| R |  | Frontal pole | 30 | 42 | 6 | 8.8* |
|  |  | Premotor cortex | 38 | 6 | 60 | 14.8* |
|  |  | Middle/inferior frontal gyrus | 60 | 0 | 44 | 7.4* |
|  |  | Insula | 38 | 16 | 8 | 5.4 |
|  |  | Intraparietal sulcus | 40 | -42 | 44 | 6.4* |
|  |  | Visual cortex | 10 | -100 | -8 | 12.2* |
| ***Controls*** | | | | | | |
| L |  | Premotor cortex | -32 | 0 | 66 | 5.9* |
|  |  | Middle/inferior frontal gyrus | -42 | 0 | 34 | 6.6* |
|  |  | Insula | -42 | 18 | 4 | 5.3 |
|  |  | Intraparietal sulcus | -30 | -52 | 44 | 8.2* |
|  |  | Visual cortex | -24 | -94 | -2 | 9.0* |
| C |  | ACC/SMA | -6 | 12 | 54 | 6.8* |
| R |  | Premotor cortex | 28 | 4 | 56 | 6.8* |
|  |  | Middle/inferior frontal gyrus | 46 | 6 | 28 | 6.4 |
|  |  | Insula | 34 | 20 | 2 | 6.6 |
|  |  | Intraparietal sulcus | 34 | -46 | 48 | 9.0* |
|  |  | Visual cortex | 24 | -84 | 6 | 6.9* |
| * p < 0.05 FWE corrected at cluster level. | | | | | | |
| Abbreviations: L, left; R, right; C central; ACC/SMA, anterior cingulate cortex/supplementary motor area. | | | | | | |

| **Table S2. Regions recruited during the study phase of letter condition** | | | | | | |
| --- | --- | --- | --- | --- | --- | --- |
| **Hem** |  | **Maxima location** | **MNI Coordinates** | | | **T** |
| ***C.L.*** |  |  |  |  |  |  |
| L |  | Middle/inferior frontal gyrus | -52 | 0 | 46 | 21.2* |
|  |  | Premotor cortex | -26 | 0 | 46 | 4.1* |
|  |  | Intraparietal sulcus | -28 | -58 | 48 | 6.0* |
|  |  | Superior parietal lobule | -22 | -78 | 48 | 6.0* |
|  |  | Superior temporal gyrus | -66 | -40 | 6 | 10.9* |
|  |  | Visual cortex | -26 | -98 | 2 | 12.3* |
| C |  | ACC/SMA | -2 | 4 | 58 | 10.1* |
| R |  | Middle/inferior frontal gyrus | 60 | 0 | 42 | 14.8* |
|  |  | Visual cortex | 40 | -88 | -8 | 12.0* |
| ***Controls*** | | | | | | |
| L |  | Premotor cortex | -28 | -6 | 62 | 5.8* |
|  |  | Middle/inferior frontal gyrus | -48 | 2 | 50 | 6.1* |
|  |  | Interparietal sulcus | -30 | -54 | 48 | 8.4* |
|  |  | Putamen | -20 | 2 | 10 | 6.6* |
|  |  | Insula | -28 | 20 | 4 | 4.5* |
|  |  | Visual cortex | -26 | -96 | -2 | 12.7* |
| C |  | ACC/SMA | -4 | 4 | 60 | 8.5* |
| R |  | Middle/inferior frontal gyrus | 58 | -8 | 50 | 5.6 |
|  |  | Intraparietal sulcus | 36 | -52 | 52 | 5.4* |
|  |  | Insula | 28 | 38 | 2 | 5.6 |
|  |  | Visual cortex | 30 | -94 | 2 | 8.3* |
| * p < 0.05 FWE corrected at cluster level. | | | | | | |
| Abbreviations: L, left; R, right; C central; ACC/SMA, anterior cingulate cortex/supplementary motor area. | | | | | | |

| **Table S3. Regions recruited during the study phase of 1-digit condition** | | | | | | |
| --- | --- | --- | --- | --- | --- | --- |
| **Hem** |  | **Maxima location** | **MNI Coordinates** | | | **T** |
| ***C.L.*** | | | | | | |
| L |  | Middle/inferior frontal gyrus | -54 | 0 | 46 | 12.6* |
|  |  | Superior temporal gyrus | -68 | -40 | 12 | 6.7 |
|  |  | Visual cortex | -26 | -100 | 2 | 9.5* |
| C |  | ACC/SMA | -2 | 6 | 56 | 5.7* |
| R |  | Middle/inferior frontal gyrus | 60 | -2 | 46 | 8.3* |
|  |  | Visual cortex | 18 | -102 | -4 | 6.9* |
| ***Controls*** | | | | | | |
| L |  | Middle/inferior frontal gyrus | -40 | -2 | 32 | 7.2* |
|  |  | Intraparietal sulcus | -32 | -56 | 52 | 6.2 |
|  |  | Visual cortex | -26 | -96 | -2 | 11.1* |
| C |  | ACC/SMA | -2 | 4 | 60 | 8.6* |
| R |  | Visual cortex | 34 | -92 | 2 | 6.4* |
| * p < 0.05 FWE corrected at cluster level. | | | | | | |
| Abbreviations: L, left; R, right; C central; ACC/SMA, anterior cingulate cortex/supplementary motor area. | | | | | | |

| **Table S4. Regions recruited during the recall phase of 2-digit condition** | | | | | | |
| --- | --- | --- | --- | --- | --- | --- |
| **Hem** |  | **Maxima location** | **MNI Coordinates** | | | **T** |
| ***C.L.*** | | | | | | |
| L |  | Frontal pole | -36 | 44 | 10 | 9.8* |
|  |  | Premotor cortex | -38 | 2 | 36 | 10.3* |
|  |  | Middle/inferior frontal gyrus | -52 | 8 | 38 | 10.1* |
|  |  | Intraparietal sulcus | -38 | -32 | 64 | 12.0* |
|  |  | Superior parietal lobule | -20 | -62 | 66 | 10.2* |
|  |  | Insula | -32 | 18 | 6 | 9.5* |
|  |  | Thalamus | -12 | -18 | 8 | 8.4* |
|  |  | Primary sensory/motor cortex | -34 | -32 | 42 | 9.7* |
|  |  | Superior temporal gyrus | -68 | -40 | 12 | 5.8* |
|  |  | Putamen | -22 | 4 | 0 | 3.7* |
|  |  | Visual cortex | -14 | -80 | -12 | 9.3* |
| C |  | ACC/SMA | -4 | 16 | 44 | 10.1* |
| R |  | Frontal pole | 30 | 44 | 4 | 10.5* |
|  |  | Premotor cortex | 28 | 8 | 50 | 8.0* |
|  |  | Middle/inferior frontal gyrus | 60 | 14 | 20 | 8.7* |
|  |  | Intraparietal sulcus | 38 | -60 | 40 | 9.2* |
|  |  | Insula | 38 | 20 | -4 | 8.6* |
|  |  | Thalamus | 12 | -14 | 10 | 5.3* |
|  |  | Primary sensory/motor cortex | 68 | -14 | 26 | 8.0* |
|  |  | Visual cortex | 8 | -94 | -6 | 13.2* |
| ***Controls*** | | | | | | |
| L |  | Frontal pole | -38 | 48 | 4 | 7.4* |
|  |  | Premotor cortex | -30 | -4 | 60 | 10.1* |
|  |  | Middle/inferior frontal gyrus | -52 | 14 | 36 | 12.9* |
|  |  | Intraparietal sulcus | -36 | -52 | 46 | 14.9* |
|  |  | Insula | -32 | 18 | -2 | 9.7* |
|  |  | Thalamus | -22 | -16 | 0 | 5.8* |
|  |  | Primary sensory/motor cortex | -48 | -38 | 50 | 9.8* |
|  |  | Visual cortex | -24 | -92 | -8 | 8.5* |
| C |  | ACC/SMA | -2 | 22 | 48 | 9.5* |
| R |  | Frontal pole | 30 | 56 | 0 | 6.3 |
|  |  | Premotor cortex | 34 | 4 | 56 | 6.9* |
|  |  | Middle/inferior frontal gyrus | 46 | 10 | 32 | 7.3* |
|  |  | Intraparietal sulcus | 30 | -64 | 50 | 10.9* |
|  |  | Insula | 34 | 24 | -6 | 9.5* |
|  |  | Thalamus | 16 | -14 | 10 | 5.9* |
|  |  | Primary sensory/motor cortex | 44 | -36 | 42 | 7.5* |
|  |  | Visual cortex | 24 | -86 | -12 | 8.9* |
| * p < 0.05 FWE corrected at cluster level. | | | | | | |
| Abbreviations: L, left; R, right; C central; ACC/SMA, anterior cingulate cortex/supplementary motor area. | | | | | | |

| **Table S5. Regions recruited during the recall phase of letter condition** | | | | | | |
| --- | --- | --- | --- | --- | --- | --- |
| **Hem** |  | **Maxima location** | **MNI Coordinates** | | | **T** |
| ***C.L.*** | | | | | | |
| L |  | Frontal pole | -36 | 46 | 10 | 6.9* |
|  |  | Premotor cortex | -30 | -14 | 68 | 14.7* |
|  |  | Middle/inferior frontal gyrus | -54 | 6 | 38 | 13.1* |
|  |  | Intraparietal sulcus | -28 | -64 | 54 | 12.0* |
|  |  | Superior parietal lobule | -18 | -76 | 52 | 12.6* |
|  |  | Insula | -32 | 18 | 6 | 8.9* |
|  |  | Thalamus | -14 | -22 | 8 | 9.3* |
|  |  | Primary sensory/motor cortex | -38 | -34 | 64 | 14.5* |
|  |  | Superior temporal gyrus | -66 | -40 | 14 | 7.0* |
|  |  | Putamen | -26 | -6 | 6 | 7.0* |
|  |  | Visual cortex | -8 | -82 | -2 | 10.9* |
| C |  | ACC/SMA | -2 | 0 | 48 | 12.0* |
| R |  | Frontal pole | 30 | 44 | 4 | 9.3* |
|  |  | Premotor cortex | 38 | 0 | 58 | 11.1* |
|  |  | Middle/inferior frontal gyrus | 60 | 10 | 30 | 10.2* |
|  |  | Intraparietal sulcus | 36 | -58 | 40 | 8.3* |
|  |  | Insula | 34 | 14 | 6 | 6.1* |
|  |  | Thalamus | 12 | -18 | 8 | 5.1* |
|  |  | Primary sensory/motor cortex | 46 | -40 | 56 | 8.8* |
|  |  | Visual cortex | 22 | -92 | -12 | 5.6* |
| ***Controls*** | | | | | | |
| L |  | Frontal pole | -38 | 48 | 8 | 6.4 |
|  |  | Premotor cortex | -30 | -2 | 60 | 11.3* |
|  |  | Middle/inferior frontal gyrus | -40 | 2 | 32 | 8.6* |
|  |  | Intraparietal sulcus | -34 | -54 | 48 | 23.1* |
|  |  | Insula | -34 | 18 | 6 | 6.6* |
|  |  | Thalamus | -24 | -24 | 6 | 6.7* |
|  |  | Primary sensory/motor cortex | -48 | -26 | 42 | 12.1* |
|  |  | Visual cortex | -16 | -92 | 2 | 11.7* |
| C |  | ACC/SMA | 0 | 12 | 48 | 12.2* |
| R |  | Premotor cortex | 28 | 12 | 58 | 8.4* |
|  |  | Middle/inferior frontal gyrus | 48 | 12 | 30 | 6.0* |
|  |  | Intraparietal sulcus | 28 | -64 | 48 | 9.0* |
|  |  | Insula | 36 | 30 | 0 | 6.9* |
|  |  | Thalamus | 18 | -6 | 0 | 6.5* |
|  |  | Primary sensory/motor cortex | 54 | -30 | 54 | 8.7* |
| * p < 0.05 FWE corrected at cluster level. | | | | | | |
| Abbreviations: L, left; R, right; C central; ACC/SMA, anterior cingulate cortex/supplementary motor area. | | | | | | |

| **Table S6. Regions recruited during the recall phase of 1-digit condition** | | | | | | |
| --- | --- | --- | --- | --- | --- | --- |
| **Hem** |  | **Maxima location** | **MNI Coordinates** | | | **T** |
| ***C.L.*** | | | | | | |
| L |  | Premotor cortex | -28 | -16 | 72 | 13.5* |
|  |  | Middle/inferior frontal gyrus | -56 | 8 | 34 | 12.5* |
|  |  | Intraparietal sulcus | -28 | -64 | 52 | 10.5* |
|  |  | Superior parietal lobule | -14 | -78 | 54 | 11.1* |
|  |  | Insula | -34 | 18 | 8 | 6.1* |
|  |  | Thalamus | -14 | -20 | 8 | 6.7* |
|  |  | Primary sensory/motor cortex | -58 | -24 | 46 | 13.4* |
|  |  | Superior temporal gyrus | -56 | -58 | 10 | 5.0* |
|  |  | Putamen | -22 | 0 | 8 | 5.5* |
|  |  | Visual cortex | -8 | -82 | -2 | 10.3* |
| C |  | ACC/SMA | -2 | 0 | 50 | 10.4* |
| R |  | Premotor cortex | 40 | 0 | 58 | 9.7* |
|  |  | Middle/inferior frontal gyrus | 62 | 10 | 30 | 11.2* |
|  |  | Intraparietal sulcus | 36 | -60 | 40 | 5.6* |
|  |  | Thalamus | 12 | 2 | -12 | 6.2* |
|  |  | Primary sensory/motor cortex | 48 | -38 | 58 | 8.6* |
|  |  | Visual cortex | 42 | -86 | -6 | 5.3* |
| ***Controls*** | | | | | | |
| L |  | Premotor cortex | -28 | -4 | 58 | 8.4* |
|  |  | Middle/inferior frontal gyrus | -54 | 6 | 26 | 13.1* |
|  |  | Intraparietal sulcus | -36 | -52 | 48 | 13.3* |
|  |  | Insula | -34 | 18 | 6 | 6.4* |
|  |  | Thalamus | -24 | -18 | 2 | 8.3* |
|  |  | Primary sensory/motor cortex | -44 | -30 | 50 | 9.6* |
|  |  | Visual cortex | -10 | -86 | 6 | 8.0* |
| C |  | ACC/SMA | -8 | 6 | 54 | 7.3* |
| R |  | Premotor cortex | 28 | 12 | 58 | 5.7* |
|  |  | Middle/inferior frontal gyrus | 56 | 10 | 16 | 8.8* |
|  |  | Intraparietal sulcus | 24 | -64 | 48 | 8.4* |
|  |  | Insula | 42 | 20 | 4 | 5.8* |
|  |  | Primary sensory/motor cortex | 58 | -30 | 54 | 8.0* |
|  |  | Visual cortex | 14 | -84 | 10 | 10.1* |
| * p < 0.05 FWE corrected at cluster level. | | | | | | |
| Abbreviations: L, left; R, right; C central; ACC/SMA, anterior cingulate cortex/supplementary motor area. | | | | | | |

| **Table S7 Direct comparison between C.L. and controls during the recall phase of 2-digit condition vs letter condition** | | | | | | |
| --- | --- | --- | --- | --- | --- | --- |
| **Hem** |  | **Maxima location** | **MNI Coordinates** | | | **T** |
| ***C.L. (2-digit - letter)> Controls(2-digit - letter)*** | | | | | | |
| L |  | Frontal Pole | -36 | 38 | 4 | 18.0* |
|  |  | Premotor cortex | -38 | 14 | 46 | 17.7* |
|  |  | Insula | -32 | 16 | 22 | 16.1* |
|  |  | Superior parietal lobule | -36 | -52 | 38 | 18.7* |
|  |  | Superior parietal lobule | -38 | -64 | 56 | 8.7 |
|  |  | Basal ganglia | -8 | 10 | 6 | 6.0 |
|  |  | Parietooccipital sulcus | -22 | -66 | 18 | 10.7* |
| C |  | ACC/SMA | -6 | 26 | 36 | 10.3* |
| R |  | Frontal pole | 34 | 52 | -2 | 8.2* |
|  |  | Superior frontal gyrus | 36 | 38 | 32 | 7.6 |
| C |  | Cerebellum/midbrain | 10 | -46 | -34 | 10.4* |
| ***C.L. (2-digit - letter)< Controls(2-digit - letter)*** | | | | | | |
| L |  | Intraparietal sulcus | -42 | -42 | 42 | 11.2* |
|  |  | Middle frontal gyrus | -58 | -14 | 30 | 11.2* |
|  |  | M/IFG/Insula | -48 | 6 | -4 | 7.8* |
|  |  | Visual cortex | -34 | -70 | -10 | 14.2* |
|  |  | Cerebellum | -40 | -56 | -26 | 6.4* |
| R |  | Intraparietal sulcus | -42 | -42 | 42 | 11.2* |
|  |  | Insula | 48 | 6 | -2 | 8.5* |
|  |  | Visual cortex | 36 | -64 | -8 | 9.8* |
|  |  | Cerebellum | 38 | -48 | -28 | 7.6* |
|  |  | Premotor cortex | 22 | -6 | 52 | 8.1* |
|  |  | Thalamus | 22 | -10 | 0 | 7.2* |

p < 0.001, uncorrected, spatial extent > 100 voxels, masked by the combined activation maps during retrieval of 2-number pairs of C.L. and of controls. Abbreviations: L, left; R, right; C, central; ACC/SMA, anterior cingulate cortex/supplementary motor area; M/IFG, middle/inferior frontal gyri. * FWE corrected at cluster level.
